# Sexual (Risk) Behavior and Risk-Reduction Strategies of Home-Based Male Sex Workers Who Have Sex with Men (MSW–MSM) in The Netherlands: A Qualitative Study

**DOI:** 10.1007/s10508-023-02648-w

**Published:** 2023-07-07

**Authors:** Charlotte M. M. Peters, Ymke J. Evers, Nicole H. T. M. Dukers-Muijrers, Christian J. P. A. Hoebe

**Affiliations:** 1 Department of Social Medicine, Care and Public Health Research Institute, Maastricht University/Maastricht UMC+, PO Box 616, 6200 MD Maastricht, The Netherlands; 2 Department of Sexual Health, Infectious Diseases and Environmental Health, Living Lab Public Health, South Limburg Public Health Service, Heerlen, The Netherlands; 3 Department of Health Promotion, Care and Public Health Research Institute, Maastricht University/Maastricht UMC+, Maastricht, The Netherlands; 4 Department of Medical Microbiology, Infectious Diseases and Infection Prevention, Care and Public Health Research Institute, Maastricht University Medical Centre, Maastricht, The Netherlands

**Keywords:** Male sex workers who have sex with men, STI/HIV, Condom use, Sexual risk behavior, Hepatitis B, Risk-reduction strategies

## Abstract

An understanding of sexual (risk) behavior is necessary to successfully develop prevention and care strategies for the sexually transmitted infections (STI) high-risk group of male sex workers who have sex with men (MSW–MSM). However, limited scientific knowledge is available on sexual (risk) behavior of (home-based) MSW–MSM. This study aimed to gain an understanding of sexual (risk) behavior, factors influencing sexual (risk) behavior, and applied risk-reduction strategies of home-based MSW–MSM. For this qualitative study, semi-structured individual interviews were conducted with 20 home-based MSW–MSM in the Netherlands. The interviews’ recordings were transcribed verbatim and thematically analyzed with Atlas.ti 8. Condom use was reported to be high during anal sex, but low during oral sex and mostly determined by STI risk perception, trust in clients, and sexual pleasure. Many experienced condom failure, while few knew what to do after condom failure and were aware of post-exposure prophylaxis (PEP). Many MSW–MSM had chemsex in the past 6 months in order to loosen up and enhance sexual pleasure. Some were not vaccinated against hepatitis B virus (HBV), mainly due to the lack of information and awareness of HBV vaccination and low risk perception of HBV. The results of this study can be used to tailor future STI/HIV risk-reduction strategies for home-based MSW–MSM and to increase awareness and uptake of available STI/HIV prevention strategies such as P(r)EP and HBV vaccination.

**Supplementary Information:**

The online version contains supplementary material available at 10.1007/s10508-023-02648-w.

## Introduction

Globally, male sex workers who have sex with men (MSW–MSM) have shown to have a higher risk for acquiring sexually transmitted infections (STI), including human immunodeficiency virus (HIV), in comparison with female sex workers (FSW) and (non-sex worker) men who have sex with men (MSM) (Baral et al., 2015). We define MSW–MSM as men who have sex with men in exchange for money or goods. A meta-analysis among 55 countries has shown that MSW–MSM overall were significantly more likely to have been diagnosed with an STI in the past 12 months (aOR 1.75) and to ever have been diagnosed with HIV (OR 1.30–aOR 1.60) compared to MSM (Berg et al., 2019; Oldenburg et al., 2015). Studies in Western Europe, The Netherlands and Belgium, have also presented higher STI positivity rates (31–46%) and newly diagnosed HIV infections (6.1–10%) in MSW–MSM compared to FSW (STI: 9–24%; HIV: 0–2%) (Drückler et al., 2020; Leuridan et al., 2005; Verhaegh-Haasnoot et al., 2015). New hepatitis B virus (HBV) infections were determined in 5–14% of MSW–MSM (FSW: 0%; MSM: 0%) and 58–71% were not (fully) HBV-vaccinated, compared to 48% of FSW and 35% of MSM (Leuridan et al., 2005; Verhaegh-Haasnoot et al., 2015). Furthermore, MSW–MSM seem to be more likely to display sexual risk behavior compared to MSM and FSW. Substance use before/during sex is reported to be higher among MSW–MSM compared to MSM and FSW (Berg et al., 2019; Drückler et al., 2020; Mgbako et al., 2019a, 2019b; Weber et al., 2001). A systematic review also indicated that 23% of the internet-based MSW–MSM who engaged in anal sex reported condomless sex (Passos & Almeida-Santos, 2020). Additionally, multiple studies have shown that a large part (43–60%) of the studied MSW–MSM population (also) had sex with women, thereby forming a bridge population in STI/HIV transmission to female sex partners (Berg et al., 2019; Drückler et al., 2020; Verhaegh-Haasnoot et al., 2015; Weber et al., 2001).

An estimated 40 to 75 percent of MSW–MSM solicits clients on the internet and MSW–MSM allegedly mostly work home-based (Donovan et al., 2010; Scott et al., 2021; Van Gelder, 2014). Soliciting clients independently of a third party from home as an in-call or out-call escort is often referred to as home-based sex work (Aidsfonds, 2018). Since many home-based sex workers mostly solicit clients on the internet, they can also be referred to as (self-employed) internet-based sex workers.

Home-based MSW–MSM are considered hidden to care, due to stigma and their internet-based working method causing individualization and thus getting in contact with healthcare has become up to MSW–MSM (Baral et al., 2015; Gelder, 2011). Despite their high STI/HIV burden, MSW–MSM have been neglected in research (Baral et al., 2015). As presented above, several studies have quantitatively assessed sexual risk behavior for MSW–MSM, but often no distinction is made between sex work locations. This distinction in work location is relevant, as the degree of STI/HIV risk for MSW–MSM depends on work context and as well as the effectiveness of subsequent public health interventions (Salhaney et al., 2021). A study conducted in the United States of America (USA) suggests that internet-based MSW–MSM their sexual (risk) behavior could be more similar to those of non-sex worker MSM compared to street-based MSW–MSM (Bimbi & Parsons, 2005). A study among Dutch FSW, however, showed that home-based FSW were significantly more likely to be diagnosed with chlamydia and gonorrhea and had a substantially lower condom use compared to window sex workers (van Dulm et al., 2020). Little is, however, known about home-based MSW–MSM their sexual (risk) behavior, factors influencing MSW–MSM’s sexual (risk) behavior and risk-reduction strategies in Western Europe. Insight into sexual (risk) behavior and risk-reduction strategies and the rationale and factors underlying this behavior, is important to tailor SHS.

To gain a deeper understanding of sexual (risk) behavior, factors influencing sexual (risk) behavior and applied risk-reduction strategies of home-based MSW–MSM, we conducted a qualitative descriptive study for which semi-structured in-depth interviews were held. This study is reported in accordance with the consolidated criteria for reporting qualitative studies (COREQ) checklist (Tong et al., 2007).

The research team comprised of both academic scholars and medical professionals with extensive prior experience in the sexual health field working at the Public Health Service South Limburg. One of the SHS providers in the Netherlands is the Public Health Service’s outpatient STI clinic (hereafter named: STI clinic). The STI clinic provides free-of-charge, anonymous and confidential SHS to STI high-risk groups, including sex workers. The SHS include provision of STI/HIV testing, hepatitis B vaccinations, pre-exposure prophylaxis (PrEP) and sexual health counseling. The STI clinic offers free hepatitis B vaccinations to MSM and sex workers. During the time of the interviews, PrEP had only recently become widely available. One can visit the STI clinic if one has a Dutch residential address.

A study previously conducted by our study group showed a high STI/HIV positivity among internet-based MSW–MSM, asking for an in-depth understanding of MSW–MSM’s sexual (risk) behavior (Verhaegh-Haasnoot et al., 2015). Two female team members, a researcher and a nurse who was also involved in the participant recruitment, conducted the interviews. Participants were informed of the interviewers’ professions, the reasons for conducting the interviews, and the main study goal.

## Method

### Participants

Participants were eligible for inclusion in this study if (1) biologically of the male sex, (2) aged 18 years and above, (3) had at least once sex with a man in exchange for money or goods in the past 6 months, (4) the sex work has taken place in the Dutch province of Limburg. Recruitment was done through purposive sampling place from November 2018 to June 2019. The participants were recruited via three routes: the STI clinic South Limburg, internet fieldwork and two male saunas. We put more emphasis on recruitment through IFW in an effort to recruit MSW–MSM who were unfamiliar with the STI clinic. The recruitment posters and the information letters were developed in Dutch, English and German to also attempt recruitment of non-Dutch sex workers. Recruitment posters were hung up in the STI clinic’s waiting rooms and in two gay saunas. Additionally, during STI consultations nurses recruited MSW–MSM and their peers. Four online platforms commonly used by MSW–MSM (Grindr, Bullchat, Boys4U and Kinky) were used for participant recruitment through internet fieldwork. Both an active (a recruitment message was sent to profiles likely of MSW–MSM) and a passive (an STI clinic nurse was present on online platforms and the recruitment poster was used as the profile picture) approach were used for recruitment. Recruitment continued until data saturation was reached. In total 29 participants were recruited, of which we interviewed 20 participants, 7 were no-shows and 2 were excluded due to not wanting to comply with the interview criteria such as interview location and due to not being a sex worker but a sex work client. Of the 20 interviewed participants, 15 were recruited through internet fieldwork, 3 through the STI clinic and 2 through the male saunas.

### Procedure and Measures

A theory-informed interview guide was developed based on relevant theoretical constructs from the health behavior models Health Belief Model (HBM) (Rosenstock, 1974) and Reasoned Action Approach Model (RAAM) (Fishbein & Ajzen, 2011), concepts retrieved from literature and themes of interest. HBM describes that health behavior is determined by perceived severity and perceived susceptibility of the health issue and benefits and barriers of the health behavior and a cue to action could provide an extra stimulant. Perceived (social) norm, attitude and knowledge might also influence health behavior as described in RAAM. We assessed factors influencing sexual (risk) behavior of MSW–MSM partly by assessing determinants of health behavior described in HBM and RAAM (Fishbein & Ajzen, 2011; Rosenstock, 1974) as well as by identifying new concepts in the interviews. Themes included were sexual practices, sexual behavior, e.g., condom use, factors influencing sexual behavior and hepatitis B vaccination. The interview guide can be found in Supplement 1.

The interviews were approximately 1.5 hours and were conducted face-to-face at a location and time of the participant’s preference. Of the 20 interviews (17 in Dutch and 3 in English), 15 were held at the STI clinic and 5 at the participant’s home. No non-participants were present at the interviews. Both oral and written informed consent were obtained. Participants received 50 euros cash as compensation for their time and a goodie bag with condoms, sexual health information leaflets and personal care products. As part of our regular outreach care, participants were offered the possibility to do an STI test and to receive a hepatitis B vaccination and information at the interview location or to make an appointment at the STI clinic.

The interviews were recorded by a voice recorder, transcribed verbatim and anonymized. Field notes were made after the interview regarding the general impression of the interviews. Transcripts were not returned to the participants for comments.

### Data Analysis

For this study, we performed a thematic analysis, allowing us to identify, analyze, organize and report themes in the data in detail (Braun & Clarke, 2006). To ensure the quality of our thematic analysis, we used the 15-point checklist of criteria for good thematic analysis developed by Braun and Clarke.

All transcripts were imported into the software Atlas.ti 8. We used a hybrid process of inductive and deductive coding. An initial coding structure was developed based on concepts and themes from the interview guide. Emergent codes were added based on the inductive coding strategy of active reading. Coding was done by the researcher who had also conducted the interviews. The first two coded transcripts were reviewed and coded by a senior researcher and discussed by the two coders until an inter-coder agreement was reached. Finally, the code descriptions were discussed with the research team for interpretation purposes.

## Results

A diverse group of 20 male sex workers who have sex with men participated in this study (Table 1). All participants were assigned the male sex at birth. Some of those unemployed besides their sex work were still in education, unable to work due to medical reasons or retired. The duration of sex work ranged from 3 months to 32 years; however, sex work was often done with intermissions. At the time of the interview 7, participants were in a relationship, of which 2 had a relationship with a man. Nearly half (45%) of the participants identified as bisexual or heterosexual. Those who identified as heterosexual had a male gender identity.

**Table 1:** Sociodemographic characteristics of the study population

Characteristic	*N* (%)
*Sex assigned at birth*	
Male	20 (100)
*Gender identity*	
Male	18 (90)
Female	2 (10)
Average age (in years)	39.9 (Range: 18–66; Median: 39.5)
*Country of birth*^1^	
The Netherlands	14 (70)
Western Europe other	3 (15)
Eastern Europe	2 (10)
South America	1 (5)
*Level of education*^*2*^	
Low	2 (10)
Medium	10 (50)
High	8 (40)
*Employment besides sex work*	
Employed	11 (55)
Unemployed	9 (45)
Duration of sex work (range)	3 months–32 years
*Relationship status*	
In a relationship	7 (35)
Not in a relationship	13 (65)
*Sexual preference*	
Homosexual	11 (55)
Bisexual	7 (35)
Heterosexual	2 (10)

^1^According to United Nations Statistics Division geographic regions (UNSD, 2021)

^2^Level of education was categorized into low: elementary, pre-vocational secondary; medium: senior general secondary, pre-university, secondary vocational; high: higher professional, university

### Sexual Behavior

#### Sexual Acts

The majority of the sex workers reported having oral and manual sex with their clients. Not all MSW–MSM had anal sex during sex work due to health risks and not enjoying anal sex. Many participants who did have anal sex, only had insertive anal sex during sex work and some had receptive anal sex. Only a few were versatile during sex work, while some who solely had insertive anal sex during sex work were versatile during sex in a private setting. Those who do not have anal sex during sex work often did sex work in the form of “soft sex” (i.e., manual and oral sex) or performed an erotic massage. A couple of participants also used toys, engaged in rimming (oral-anal sex), BDSM (bondage, discipline, sadism, masochism), sex involving urine and had threesomes during sex work. One MSW–MSM sometimes also had vaginal sex with a female sex partner during sex work.

It's really the massaging and jerking off. A step higher, then oral is added. Because most men want to blow me. I don't think it's gross, so I'll allow it. And if I really like someone I’ll blow him too. And every now and then comes anal. But then I'll be top. – P6, 55y.

The MSW–MSM often clearly indicated in advance what sexual acts they would and wouldn’t perform during sex work (e.g., swallowing semen). However, many would also base their sexual acts during sex work on the trust in and connection with the client. The trustworthiness and health of the client was assessed based on the physical appearance and behavior of the client, and sometimes as well on proof of a recent STI test.

A lot of people ask certain sex things, what do you do, what don't you do? But I always find that so difficult to say, it depends on the person. You don't know what you- I could say I'm doing this and that and that, but if the person doesn't feel right, then that thing won't happen. – P1, 39y.

#### Sex Work Intensity

The work intensity of sex work varied from daily sex work to four times a year, with the majority doing sex work 1 to 5 times a week often with one client at a time. Participants determined their sex work intensity on convenience, sexual drive, the need for money and the demand for their services. One participant was motivated to limit his clients to limit his exposure to STI and protect his sexual health.

It primarily depends on if I feel like it, but sometimes I just don’t have any requests at all. – P10, 50y.

Few participants did daily sex work with the intention to get as many clients as possible. One participant planned 2 to 3 nights a month to do sex work for an entire night with 4 to 7 clients. The intensity of sex work had decreased for a couple of participants due to the strain highly intensive sex work had on their overall well-being, due to getting older and due to a decreasing demand for sex work services.

#### Chemsex

Many said to have participated in drug use before or during sex, also referred to as chemsex, in the past six months both in a private and sex work setting. Speed, GHB and XTC were mostly used, as well as cannabis, MDMA, GBL, cocaine, Tina (methamphetamine) and poppers (not included in the chemsex definition). Participants said to engage in chemsex to be able to loosen up, to be more alert and awake longer, to relieve pain during sex, to enhance pleasure and sexual functioning and by request of clients. Some often or always used cannabis, GHB, GBL or poppers during sex work, while some only occasionally used drugs during sex work, often if requested by the client.

GHB is a must during sex. I think so, GHB is, so to speak, a sex drug. It takes the edges off a bit and the advantage is that after an hour and a half it's just gone. – P9, 42y.

#### Private Condom Use

A minority of the interviewed MSW–MSM reported condom use with their private sex partner until they both tested negative for STI or reported to always use a condom with their private sex partners. Most, however, reported no condom use with private partners. Some also had condomless vaginal sex with private female partners. Reasons provided for condomless private sex were that one would trust their partner and that private sex often involves love. One participant mentioned discussing STI and safety with (chem)dates to estimate if it would be safe to have condomless sex. Another participant’s private partner had stressed the importance of having sex with a condom during sex work.

Private sex indeed is usually without. Because for example I just know that my boyfriend doesn't have an STI now. So then I feel like, okay I can't get anything from that either, or the chance is just very small that I can get something of it. – P14, 18y.

#### Sex Work Oral Condom Use

Most MSW–MSM did not use a condom during oral sex work, only if requested by the client. A couple of MSW–MSM only had condomless oral sex if clients would not ejaculate during oral sex, could show results of a recent STI test or if the client is attractive. If the sex worker did not trust the client, a condom would be used or only manual sex would be performed. Some sex workers said to always use a condom during oral sex.

Oral actually unprotected sex and yeah, so you know there's always a risk factor. If I don't trust it completely then I just don't do it or in the worst case I put on a condom or I stick to manual work and so I really stick to safety.- P10, 50y.

#### Sex Work Anal Condom Use

Anal sex was reported to be mostly done with a condom. Some said to be really strict when it comes to anal condom use, in contrast to some who reported to make exceptions. One sex worker had become more lenient toward anal condom use after starting PrEP. Many clients supposedly request condomless anal sex, a participant even mentioned that half of his requests are for condomless anal sex, which he declines. Those who use toys during their sex work reported using a condom on the toys if they are not the client’s toys.

They never have to ask me to do without, that's not going to happen. You also see that very often people say: “without a condom.” That's actually happens a lot, I'm really amazed at how many people do that. – P1, 39y.

#### Factors Influencing Condom Use

**Fig. 1:**
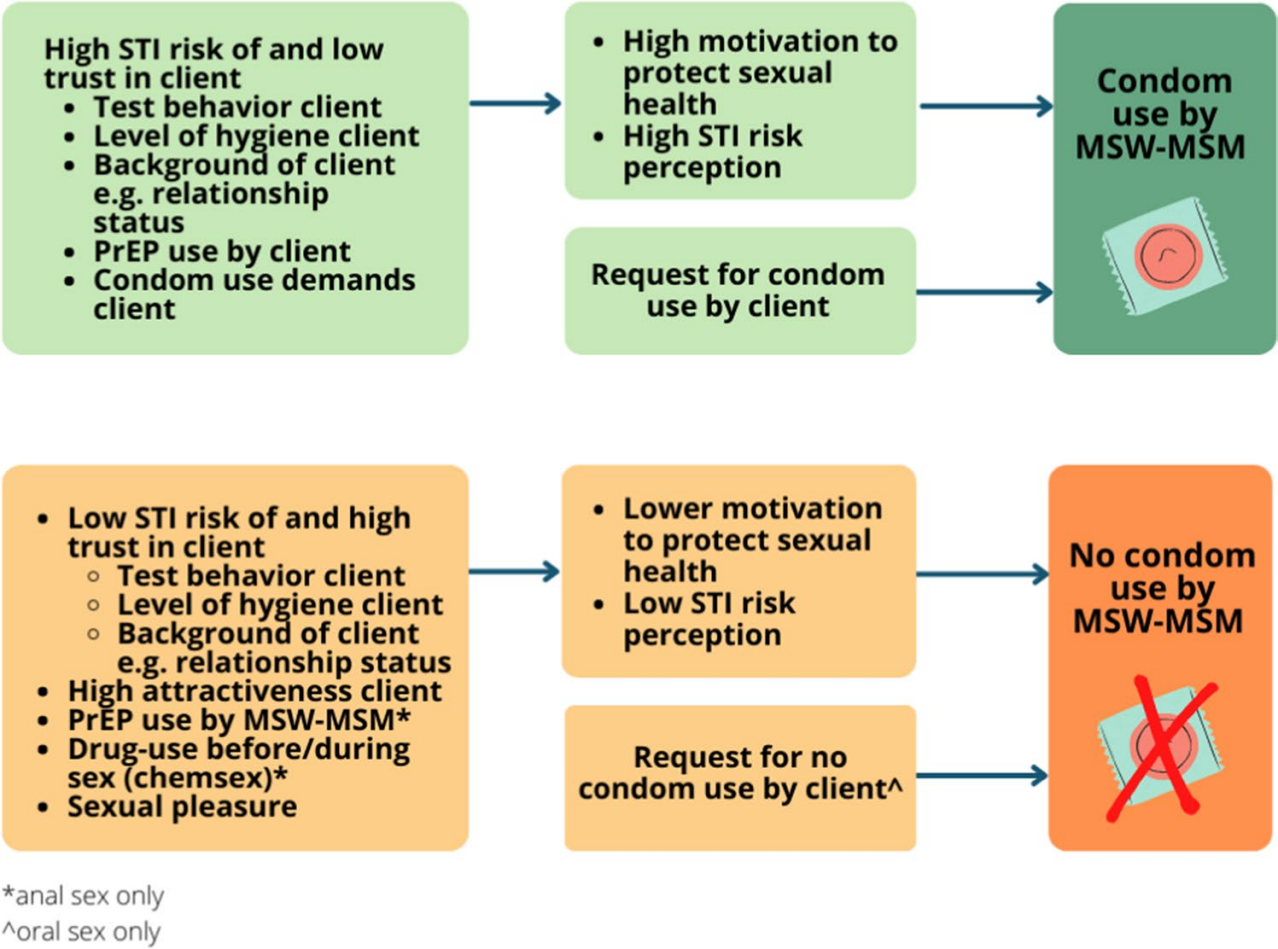
Factors influencing condom use as reported by MSW–MSM

Condom use of MSW–MSM was mostly determined by the motivation to protect their health and STI risk perception. The STI risk of a client was assessed by inquiring about test behavior of the client, the level of the client’s personal hygiene and the background of the client, e.g., if client is in a relationship. Sexual pleasure and trust in the client or sex partner were also factors influencing condom use. Some MSW–MSM also determined their condom use based on the attractiveness of their client, since they were more likely not to use a condom if attracted to the client. Condom use requests of the client were also factored into condom use (e.g., a participant wants oral condom use but clients don’t want a condom to be used during oral sex and he won’t have any clients otherwise). On the other hand, client requests for condomless anal sex were often denied by the sex workers. PrEP use by the MSW–MSM caused more lenient condom use, but PrEP use among clients did not since the sex workers often perceived such clients as a higher STI risk. Drug use also determined condom use, since one participant said to not use condoms during chemsex dates, mostly to enhance pleasure. The participants perceived the social norm of condom use by MSW–MSM in general as variable. Advertisements for condomless sex were seen online frequently, some even reporting that 70–90% is advertising with condomless sex, while on the other hand advertisements for safe sex were often seen as well. Our study population’s condom use, however, was reported not to be influenced by the perceived social norm of condom use. Factors influencing condom use behavior for oral sex and anal sex as reported by MSW–MSM are shown in Fig. 1.

Yes, actually every sexual contact I've had for money has always been with a condom. Because you don't know who it is, who he's been in bed with and it's just a really big risk. But with people who I’ve known for many years and of which I know with whom they laid in bed, with them I’m like I know they don’t have anything, so come on. But otherwise no, I’ll use a condom. – P14, 18y.

#### Condom Failure

Nearly half of the participants had previously experienced condom failure. Most of them got tested for STI after condom failure; however, one participant had the misperception that washing the anus after condom failure was sufficient to prevent STI. Only few participants were confident on what to do after condom failure and were aware of PEP.

I wouldn't know, no, I wouldn't know what to do. It luckily has not happened, but- – P16, 49y.

#### Lubricant Use

Lubricant was often or always being used by most MSW–MSM during sexual intercourse. Lubricant was mostly used to make anal sex easier, more pleasant and less painful. One sex worker, however, mentioned that the provision of lubricant is the responsibility of the client. Some did not use lubricant, mostly due to the absence of anal intercourse during sex work or due to using massage oil instead of lubricant.

Yes, the sex is better if you use lubricant. That is more of a requirement, so to speak. – P9, 42y.

#### PrEP Awareness/Knowledge

During the time of the interviews, PrEP had only recently become widely available. Many participants had little to no knowledge about PrEP. Some of them were aware of PrEP, but lacked substantive knowledge, e.g., could not explain what PrEP was for. Part of the MSW–MSM were aware of PrEP and had enough knowledge to (roughly) explain what PrEP entails. Some had read online about PrEP, heard from friends or heard from the STI clinic about PrEP.

#### PrEP Use

Many were interested in PrEP, few also were already registered on the waiting list and one participant was using PrEP at the time of the interview. Some said not to be interested in PrEP due to a lack of urgency, mostly based on their sexual behavior and absence of anal sex, but also due to a negative attitude toward PrEP and being uncertain of its trustworthiness.

I think all men should just be careful. But yes, if I can help prevent a virus or whatever it is, why not? I'd be interested. – P18, 48y.

#### Factors Influencing PrEP Use

PrEP use was determined by motivation to protect their health and be able to have safer sex, as well as fear of HIV.

Before the PrEP, I was really afraid. Yeah.…This is the reason, principal reason, I just decided to take a PrEP. – P8, 35y.

Being able to afford PrEP and coverage by health insurance were also important factors for PrEP use. Attitude toward PrEP also determined PrEP use (e.g., a participant did not want to use PrEP because of a negative attitude toward PrEP, since it was perceived as a condom substitute). One participants first wanted to obtain information on possible interactions with other medication before considering PrEP.

### Hepatitis B

#### Hepatitis B Vaccination Behavior

The greater part of the MSW–MSM were fully vaccinated against hepatitis B. In addition, one participant had yet to receive the third vaccination. Most were vaccinated at the STI clinic, others were vaccinated by the general practitioner, during their education or in prison. Part of MSW–MSM had not been vaccinated against hepatitis B.

#### Factors Influencing Hepatitis B Vaccination

Participants mainly got vaccinated against hepatitis B to protect their health and to prevent contracting and passing on hepatitis B. Many said they would take anything if it would benefit their health. Some also got hepatitis B vaccinations for traveling purposes or for their education as a health professional. Other factors of influence were a positive attitude toward the HBV vaccinations, due to the vaccinations being free-of-charge and being aware of the benefits of the vaccination, as well as perceiving hepatitis B as severe due to having a friend with hepatitis B.

I'd seen that brochure and it had all the positive things in it, I was like—And it was also free, I didn't have to pay, I’m thinking, if it's for my health, then I’ll have to do it. – P7, 39y.

**Fig. 2:**
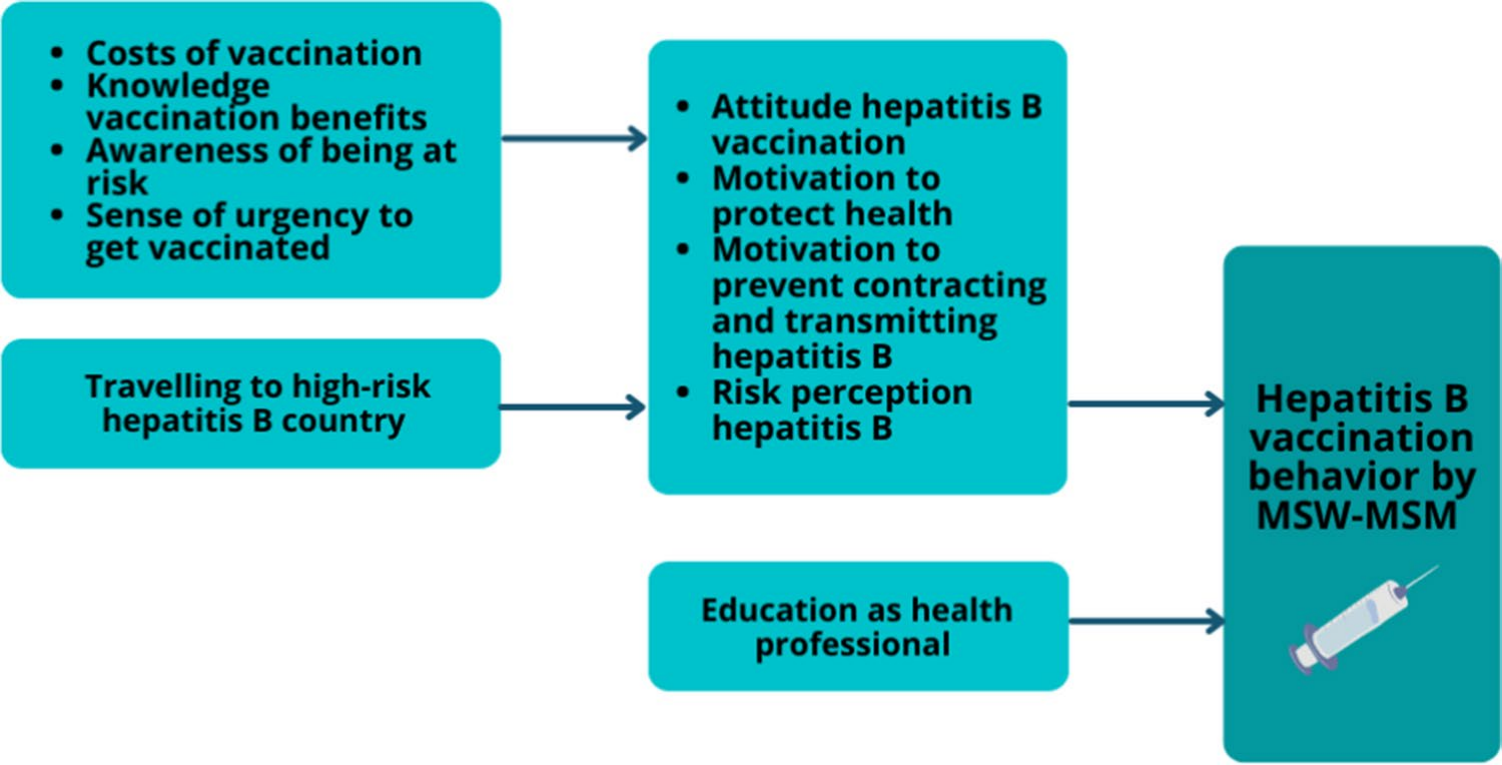
Factors influencing hepatitis B vaccination behavior as reported by MSW–MSM

Several participants were not vaccinated, mostly due to not being aware of being at risk and the need to get vaccinated, as well as a low hepatitis B risk perception and a low sense of urgency. Factors influencing hepatitis B vaccination behavior as reported by MSW–MSM are shown in Fig. 2.

Um, I haven't really thought about it. I don't consider myself at risk, let me put it that way. – P11, 66y.

## Discussion

In this study, we qualitatively assessed sexual (risk) behavior of home-based MSW–MSM and gained an understanding of MSW–MSM’s risk-reduction strategies and factors influencing sexual (risk) behavior. Our study population of MSW–MSM reported condom use during sex work to be high with anal sex, low with oral sex and mainly determined by STI risk perception, trust in client and sexual pleasure. Most MSW–MSM were unaware of what to do after condom failure, PEP and PrEP, despite many of them having experienced condom failure. Many MSW–MSM had chemsex in the past 6 months by mostly using speed, GHB and XTC in order to loosen up, be more alert, to relieve pain and enhance sexual pleasure. To protect their sexual health against STI, MSW–MSM applied risk-reduction strategies. They would avoid certain sexual acts which they perceived as risky and would adjust their sexual acts based on self-assessed trustworthiness and health of their client. This was assessed based on test behavior and sexual behavior (e.g., PrEP use and condom use requests) of the client, the level of the client’s hygiene and the background of the client such as relationship status. A part of MSW–MSM was not vaccinated against hepatitis B (HBV), mainly due to the lack of information and awareness of HBV vaccination and a low risk perception of HBV.

To our knowledge, this is the first qualitative study assessing sexual practices and sexual (risk) behavior of home-based MSW–MSM in Western Europe.

### Literature Comparison

In a quantitative Dutch study among self-employed sex workers, condom use was similarly to our study self-reported to be relatively high with anal sex and low with oral sex; however, only one-third of their MSW study population (also) had sex with men (Kampman et al., 2022). Previous qualitative research among non-sex worker MSM in the USA identified physical discomfort of condoms, relationship trust, usage based on the type of sexual act, substance use, and knowledge of a partner’s STI/HIV status as factors related to condom use (Giano et al., 2020). In the current study, drug use and trust were as well identified as factors influencing condom use and MSW–MSM partly based their trust on knowledge of their client’s STI/HIV status by inquiring for a recent STI test. Condom use in our study was also depending on the sexual act in which MSW–MSM were engaging, as they were less likely to use condoms with oral sex compared to anal sex. Trust in client was, as established in this study, also reported to be an influencing factor in condom use in a study among MSW–MSM in China, with MSW–MSM perceiving clients as “safe” when they became regulars (Kong, 2008). In our study, we, however, determined that STI risk of the client and trust in the client is assessed based on multiple factors, i.e., test behavior, level of hygiene, background and sexual behavior of the client. Individual health concerns were found to be motivational factors to avoid sexual risk behavior by agency-based MSW–MSM, as well as the lack of attractiveness of clients while the attractiveness of the client was a motivational factor to have condomless anal sex for home-based MSW–MSM in a study from the USA (Smith & Seal, 2008). These results align with our current study findings in which motivation to protect sexual health and the attractiveness of a client were identified as factors influencing condom use.

Doing sex work in isolated informal settings, such as in the home-based setting, has previously been linked to reduced control over clients, substance use and condom use (Goldenberg et al., 2015). Our home-based MSW–MSM study population, however, mostly seemed to be able to set boundaries regarding sexual acts, negotiate anal condom use to their desire and self-reported to often refuse condomless anal sex requests.

Furthermore, in our study many participants had previously experienced condom failure, but were unaware of steps to be followed after condom failure and PEP. Comparable to the results of the current study, quantitative and qualitative studies among MSM and MSW–MSM in the USA reported awareness and usage of both PEP and PrEP being low but the interest being high as well as willingness to disclose their sexual behavior to obtain PrEP (Dolezal et al., 2015; Underhill et al., 2015; Walters et al., 2017). While the majority of MSW–MSM in France were aware of PrEP and likely to use it, the actual PrEP use was low and may signify issues with access to sexual healthcare services (SHS) (Mgbako et al., 2019a, 2019b).

Similarly to our study, a quantitative Dutch study reported MSW–MSM mostly having chemsex to make sex (work) physically easier and per client request (Drückler et al., 2020). However, in our study the enhancement sexual pleasure was an additional reason to have chemsex.

Regarding HBV vaccination uptake among sex workers in Western countries, studies have mainly focused on individual characteristics and structural determinants for HBV vaccination uptake, showing the relevance of access to SHS, specialized hepatitis B vaccination (outreach) services and a personal approach (Baars et al., 2009; Mak et al., 2003; Ranjan et al., 2019; Sethi et al., 2006). Similarly to our study, determinants derived from the HBM, namely perceived susceptibility and perceived severity of HBV, were found to be (significantly) associated with HBV vaccination uptake in a quantitative study among MSM in the Netherlands, as well as perceived personal treat of HBV and the possibility that one’s lifestyle may become known (de Wit et al., 2005). Attitude of HBV vaccination, however, was not significantly associated with vaccination behavior among Dutch MSM (de Wit et al., 2005), while HBV vaccination attitude was determined to be of influence in our study among MSW–MSM.

### Strengths and Limitations

This study had several strengths. This study adds to the sparsely available scientific knowledge on home-based MSW–MSM in Western Europe. Furthermore, our recruitment method managed to successfully recruit participants from the MSW–MSM population who are considered hidden and hard to reach. We also managed to create a non-judgmental and open interview setting that enabled participants to open up about their sex life. Several limitations should be considered when interpreting this study’s results. Despite efforts to recruit a diverse and representative study population through the purposive sampling method, persons with a low education level and non-Dutch nationalities were underrepresented in our study population. This could compromise the external validity of the study results. Given the sensitive nature of the interview topics, the participants may have given socially desirable answers. Participants may have also underreported, e.g., sexual risk behavior and self-efficacy of condom use negotiation skills.

At the time of the interviews, PrEP had only recently become available. This may have influenced our study results regarding awareness and uptake of PrEP.

Due to local differences, the found study concepts might not be transferable for the entire Western European region. The sex work subculture is comparable in the border region of the Netherlands, Belgium and Germany. Considering cross-border sex work activity, concepts found in this study with regard to the MSW–MSM population and their sexual behavior might be transferable to those regions and other non-high-urban areas in Western Europe.

### Implications

This study provided insights in home-based MSW–MSM’s sexual (risk) behavior and risk-reduction strategies applied by MSW–MSM. These insights provide an opportunity to tailor STI/HIV counseling and risk-reduction strategies for home-based MSW–MSM and ensure it matches the MSW–MSM’s reality to increase STI/HIV counseling and risk-reduction strategies’ effectiveness. Future research should focus on both the development and evaluation of tailored risk-reduction strategies for (home-based) MSW–MSM.

In this study, condom failure is reported to be high as well as unawareness of PEP, indicating the MSW–MSM to be highly vulnerable of contracting HIV. Based on national STI clinic testing data from 2019 to 2021, 26.2% of MSW–MSM had ever used PrEP, compared to 21.6% of non-sex worker MSM. Given the MSW–MSM their higher vulnerability to HIV compared to non-sex worker MSM, PrEP would be a suitable biomedical intervention for MSW–MSM (Baral et al., 2015) and uptake of PrEP should be significantly higher compared to non-sex worker MSM. Future efforts of SHS should thus focus on increasing PEP and PrEP awareness and uptake among home-based MSW–MSM.

The frequently reported chemsex and associated sexual and psychosocial health risks calls for SHS to develop harm reduction programs tailored to home-based MSW–MSM. Communication efforts toward home-based MSW–MSM should focus on increasing awareness of PEP, PrEP and HBV vaccinations. For effective communication, it is, however, important to tailor communication to the needs of the MSW–MSM population (Peters et al., 2022).

### Conclusion

This study was able to qualitatively assess sexual (risk) behavior of home-based MSW–MSM and gained an understanding of MSW–MSM’s risk-reduction strategies and factors influencing sexual (risk) behavior. Despite portraying some sexual risk behavior, MSW–MSM overall seemed motivated to protect their health and the health of their clients, for which they applied risk-reduction strategies. Results of this study can be used to tailor future risk-reduction strategies for home-based MSW–MSM to reduce sexual risk behavior and STI/HIV vulnerability, as well as to increase awareness and uptake of available STI/HIV prevention strategies such as PEP and HBV vaccination.

### Supplementary Information

Below is the link to the electronic supplementary material.

Supplementary file1 (DOCX 42 kb)

## Data Availability

The data generated and analyzed during the current study are not publicly available due to the possibility of compromising individual privacy but are available from the corresponding author upon reasonable request.

## References

[CR1] Aidsfonds. (2018). *Sex work, stigma and violence in the Netherlands*. SANL-Aidsfonds. https://aidsfonds.org/resource/sex-work-stigma-and-violence-in-the-netherlands.

[CR2] Baars JE, Boon BJ, Garretsen HF, van de Mheen D (2009). Vaccination uptake and awareness of a free hepatitis B vaccination program among female commercial sex workers. Women's Health Issues.

[CR3] Baral SD, Friedman MR, Geibel S, Rebe K, Bozhinov B, Diouf D, Sabin K, Holland CE, Chan R, Cáceres CF (2015). Male sex workers: Practices, contexts, and vulnerabilities for HIV acquisition and transmission. The Lancet.

[CR4] Berg, R. C., Weatherburn, P., Marcus, U., & Schmidt, A. J. (2019). Links between transactional sex and HIV/STI-risk and substance use among a large sample of European men who have sex with men. *BMC Infectious Diseases,**19*(1). 10.1186/s12879-019-4326-3 10.1186/s12879-019-4326-3 PMC6683343 31382923

[CR5] Bimbi DS, Parsons JT (2005). Barebacking among internet based male sex workers. Journal of Gay & Lesbian Psychotherapy.

[CR6] Braun V, Clarke V (2006). Using thematic analysis in psychology. Qualitative Research in Psychology.

[CR7] de Wit JB, Vet R, Schutten M, van Steenbergen J (2005). Social-cognitive determinants of vaccination behavior against hepatitis B: An assessment among men who have sex with men. Preventive Medicine.

[CR8] Dolezal C, Frasca T, Giguere R, Ibitoye M, Cranston RD, Febo I, Mayer KH, McGowan I, Carballo-Diéguez A (2015). Awareness of post-exposure prophylaxis (PEP) and pre-exposure prophylaxis (PrEP) is low but interest is high among men engaging in condomless anal sex with men in Boston, Pittsburgh, and San Juan. AIDS Education and Prevention.

[CR9] Donovan, B., Harcourt, C., Egger, S., Schneider, K., O’Connor, J., Marshall, L., Chen, M., & Fairley, C. (2010). *The sex industry in Western Australia*. Sydney, Australia: National Centre in HIV Epidemiology and Clinical Research, The University of New South Wales.

[CR10] Drückler S, van Rooijen MS, de Vries HJ (2020). Substance use and sexual risk behavior among male and transgender women sex workers at the prostitution outreach center in Amsterdam, the Netherlands. Sexually Transmitted Diseases.

[CR11] Fishbein, M., & Ajzen, I. (2011). *Predicting and changing behavior: The reasoned action approach*. Psychology Press.

[CR12] Gelder PV (2011). Seksuele dienstverlening door mannen in Den Haag: Ontwikkelingen op het internet. Journal of Social Intervention: Theory and Practice.

[CR13] Giano Z, Kavanaugh KE, Durham AR, Currin JM, Wheeler DL, Croff JM, Hubach RD (2020). Factors associated with condom use among a sample of men who have sex with men (MSM) residing in rural Oklahoma. Journal of Homosexuality.

[CR14] Goldenberg, S. M., Duff, P., & Krusi, A. (2015). Work environments and HIV prevention: A qualitative review and meta-synthesis of sex worker narratives. *BMC Public Health, 15*(1). 10.1186/s12889-015-2491-x 10.1186/s12889-015-2491-x PMC4681074 26672756

[CR15] Kampman, C., Peters, C., Koedijk, F., Berkenbosch, T., Hautvast, J., & Hoebe, C. (2022). Sexual risk and STI testing behaviour among Dutch female and male self-employed sex workers; a cross-sectional study using an Internet based survey. *BMC Public Health,**22*(1). 10.1186/s12889-022-13582-2 10.1186/s12889-022-13582-2 PMC9185941 35681139

[CR16] Kong TS (2008). Risk factors affecting condom use among male sex workers who serve men in China: A qualitative study. Sexually Transmitted Infections.

[CR17] Leuridan E, Wouters K, Stalpaert M, Van Damme P (2005). Male sex workers in Antwerp, Belgium: A descriptive study. International Journal of STD & AIDS.

[CR18] Mak R, Traen A, Claeyssens M, Van Renterghem L, Leroux-Roels G, Van Damme P (2003). Hepatitis B vaccination for sex workers: Do outreach programmes perform better?. Sexually Transmitted Infections.

[CR19] Mgbako O, Park SH, Callander D, Brinker DA, Kuhner C, Carrico AW, Rendina HJ, Duncan DT (2019). Transactional sex, condomless anal sex, and HIV risk among men who have sex with men. International Journal of STD & AIDS.

[CR20] Mgbako O, Park SH, Mayer KH, Schneider JA, Goedel WC, Hambrick HR, Duncan DT (2019). Transactional sex and preferences for pre-exposure prophylaxis (PrEP) administration modalities among men who have sex with men (MSM). Journal of Sex Research.

[CR21] Oldenburg CE, Perez-Brumer AG, Reisner SL, Mimiaga MJ (2015). Transactional sex and the HIV epidemic among men who have sex with men (MSM): Results from a systematic review and meta-analysis. AIDS and Behavior.

[CR22] Passos TS, Almeida-Santos MA (2020). Condomless sex in Internet-based sex work: Systematic review and meta-analysis. Research, Society and Development.

[CR23] Peters, C. M. M., Dukers-Muijrers, N. H. T. M., Evers, Y. J., & Hoebe, C. J. P. A. (2022). Barriers and facilitators to utilisation of public sexual healthcare services for male sex workers who have sex with men (MSW–MSM) in The Netherlands: A qualitative study. *BMC Public Health,**22*(1). 10.1186/s12889-022-13799-1 10.1186/s12889-022-13799-1 PMC9306090 35864473

[CR24] Ranjan A, Shannon K, Chettiar J, Braschel M, Ti L, Goldenberg S (2019). Barriers and facilitators to hepatitis B vaccination among sex workers in Vancouver, Canada: Implications for integrated HIV, STI, and viral hepatitis services. International Journal of Infectious Diseases.

[CR25] Rosenstock IM (1974). Historical origins of the health belief model. Health Education Monographs.

[CR26] Salhaney P, Biello KB, Mimiaga MJ, Scott J, Grov C, Minichiello V (2021). Global epidemiology of HIV and other sexually transmitted infections among male sex workers: Emerging approaches in prevention and treatment. The Routledge handbook of male sex work, culture, and society.

[CR27] Scott JG, Grov C, Minichiello V (2021). The Routledge handbook of male sex work, culture, and society.

[CR28] Sethi G, Holden B, Greene L, Gaffney J, Ward H (2006). Hepatitis B vaccination for male sex workers: The experience of a specialist GUM service. Sexually Transmitted Infections.

[CR29] Smith MD, Seal DW (2008). Motivational influences on the safer sex behavior of agency-based male sex workers. Archives of Sexual Behavior.

[CR30] Tong A, Sainsbury P, Craig J (2007). Consolidated criteria for reporting qualitative research (COREQ): A 32-item checklist for interviews and focus groups. International Journal for Quality in Health Care.

[CR31] Underhill K, Morrow KM, Colleran C, Holcomb R, Calabrese SK, Operario D, Galárraga O, Mayer KH (2015). A qualitative study of medical mistrust, perceived discrimination, and risk behavior disclosure to clinicians by US male sex workers and other men who have sex with men: Implications for biomedical HIV prevention. Journal of Urban Health.

[CR32] UNSD (2021). Standard country or area codes for statistical use (M49).

[CR33] van Dulm E, Marra E, Kroone MM, van Dijk AE, Hogewoning AA, van der Loeff MFS (2020). Sexually transmissible infections among female sex workers in Amsterdam between 2011 and 2016: Does risk vary by work location?. Sexual Health.

[CR34] Van Gelder, P. (2014). *Boys online/Boys offline. Seksuele dienstverlening door jongens/mannen (M $ M) in Den Haag, 2009. Update internetescorts 2009–2012*. Den Haag: Shop-Den Haag.

[CR35] Verhaegh-Haasnoot, A., Dukers-Muijrers, N. H., & Hoebe, C. J. (2015). High burden of STI and HIV in male sex workers working as internet escorts for men in an observational study: A hidden key population compared with female sex workers and other men who have sex with men. *BMC Infectious Diseases,**15*(1). 10.1186/s12879-015-1045-2 10.1186/s12879-015-1045-2 PMC4517560 26220287

[CR36] Walters SM, Rivera AV, Starbuck L, Reilly KH, Boldon N, Anderson BJ, Braunstein S (2017). Differences in awareness of pre-exposure prophylaxis and post-exposure prophylaxis among groups at-risk for HIV in New York State: New York City and Long Island, NY, 2011–2013. JAIDS Journal of Acquired Immune Deficiency Syndromes.

[CR37] Weber AE, Craib KJ, Chan K, Martindale S, Miller ML, Schechter MT, Hogg RS (2001). Sex trade involvement and rates of human immunodeficiency virus positivity among young gay and bisexual men. International Journal of Epidemiology.

