# Correction to: Sexual (Risk) Behavior and Risk-Reduction Strategies of Home-Based Male Sex Workers Who Have Sex with Men (MSW–MSM) in The Netherlands: A Qualitative Study

**DOI:** 10.1007/s10508-023-02671-x

**Published:** 2023-07-28

**Authors:** Charlotte M. M. Peters, Ymke J. Evers, Nicole H. T. M. Dukers-Muijrers, Christian J. P. A. Hoebe

**Affiliations:** 1 Department of Social Medicine, Care and Public Health Research Institute, Maastricht University/Maastricht UMC+, PO Box 616, 6200 MD Maastricht, The Netherlands; 2 Department of Sexual Health, Infectious Diseases and Environmental Health, Living Lab Public Health, South Limburg Public Health Service, Heerlen, The Netherlands; 3 Department of Health Promotion, Care and Public Health Research Institute, Maastricht University/Maastricht UMC+, Maastricht, The Netherlands; 4 Department of Medical Microbiology, Infectious Diseases and Infection Prevention, Care and Public Health Research Institute, Maastricht University Medical Centre, Maastricht, The Netherlands

**Correction to: Archives of Sexual Behavior** 10.1007/s10508-023-02648-w

The following Acknowledgments should be noted for the article.

