# Supplementary material for: Sexual (Risk) Behavior and Risk-Reduction Strategies of Home-Based Male Sex Workers Who Have Sex with Men (MSW–MSM) in The Netherlands: A Qualitative Study
Source: Arch Sex Behav. 2023 Jul 7;52(8):3329–39. doi: 10.1007/s10508-023-02648-w (PMC10703956; doi:10.1007/s10508-023-02648-w)
Supplement: Supplementary file 1 — Supplementary file1 (DOCX 42 kb) [file 10508_2023_2648_MOESM1_ESM.docx]

**Supplement 1. Interview guide for semi-structured interviews with male sex workers who have sex with men (MSW-MSM)**

**Introduction**

- Introduction of two interviewers
- The purpose of the interview and research is to gain more insight into the working methods and organization of the men who have sex with men for money and goods. In addition, we would like to investigate which factors influence whether or not you take an STI test and whether you get vaccinated against hepatitis B. We would also like to know how you can and want to be approached by the public health services’ STI clinic. Ultimately, we want to better adapt our care to your needs.
- The nurse will mainly ask you questions, the researcher occasionally supplements with questions. There are no right or wrong answers, we are especially curious about your experiences and opinion.
- Have you read the information letter about the study? If not, let the participant read and/or read it for participant.
- Do you have any questions about the research?
- Checking inclusion and exclusion criteria:
  - Have you had sex with men at least 1 time in the past 6 months in exchange for sex or other sex in exchange?
  - Do you do this/did you do this in the province of Limburg? / Do you advertise in the province of Limburg? (if self-identifying sex worker)
  - Are you 18 years or older?
- Review the declaration of consent for participation in scientific research and have it signed.

**Demographic characteristics**

- What is your age?
- What is your highest (finished) level of education?
- What is your country of birth? (If applicable: What is the ethnicity/country of birth of your parents/family?)
- Do you have a job right now?
- What is your biological sex? Do you identify with this?
- Why did you decide to participate in this study?

**Sex work - background**

Now we would like to talk to you further about your experiences with relationships and sexuality. Are you in a relationship right now? Can you talk a little bit about that?

- What gender are you attracted to? (sexual preference)
- Who do you have sexual relationships with?

You have indicated that you sometimes have sex with men for money or some other reward. Can you tell us a bit more about that?

- What is the reason you sometimes have sex in exchange for money and goods?
- What is your experience with this?
- Do you view this as sex work? And yourself as a sex worker?

Is it okay that we call it sex work from now on?

- How long have you been doing this (sex work)?
  - Can you talk about the beginning?
- Do you think you want to continue with sex work in the future?

Could you tell us a bit about the organization of sex work?

- Where do you work?
- How often do you work?
- Where do you acquire customers?
- Do you work alone?

Could you tell me something about the customers?

**Sex work & health**

Now we would like to talk about your health, how you experience it.

Do you ever worry about your health?

- About what? What do you do about it?

Are there certain things you absolutely do or don't do during sex/when you have sexual contact?

- Why or why not?
- Does this differ for relationships vs customers and/or type of relationship/customer?
  - What do your sex partners/clients think of that?
- What do you do to stay sexually healthy?

If condom use and/or testing has not yet been (fully) discussed with the above questions:

Condom use

- Do you use condoms during your sex work?
  - Is there a difference in condom use between work and private life?
  - How do your partner/customers look at condom use?
  - How do you think other sex workers view condom use?
  - Do you always use condoms or are there exceptions?
  - Do you also use lubricant?
- Have you ever had condom failure ?
  - If so, what did you do then?
    - PEP/ STI test?
- Do you ever use drugs?
  - If yes, what drugs?
  - Before and/or during sex?
  - Why?

STI test behavior

- Have you ever done an STI test?
- How do you view taking an STI test?
- Why did you do an STI test?
- Are there things that stop you from taking an STI test?
- Are there things that encourage you to take an STI test?
- How often would you like to get tested?
- What would you need to get tested more often?
- What does your environment (other sex workers/partner/friends/family) think about STI testing?
  - If other opinion: Do you agree with them? Why or why not? How do you deal with that?

Hepatitis B vaccination

- Are you aware of the existence of hepatitis B vaccinations?
- How do you view the hepatitis B vaccination?
- What does your environment (other sex workers/partner/friends/family) think about vaccination (against hep B)?
- Have you been vaccinated for hepatitis B?
  - If yes:
    - Where did you get vaccinated?
    - What are your reasons for getting vaccinated?
  - If no:
    - Why not? Are there things stopping you from getting vaccinated?
    - What can you do / How can we encourage you to get vaccinated?

**Perception and care offer Public Health Service STI clinic**

Can you tell us what you know about the STI clinic?

- Have you ever been to the Public Health Services?
  - Where & how did you find information about the Public Health Services (STI clinic)?
  - How did you make an appointment for an STI test/vaccination?
  - Did you find the STI clinic easily accessible?
  - Why did you get tested at the STI clinic and not somewhere else (e.g. gp or home test)
  - How many sex workers do you think have experience with the STI clinic? How do you think other sex workers view the STI clinic?

What do you think of the STI clinic?

- How do you experience contact with the nurse? Why?
- Would you like to get tested again at the STI clinic? Or rather somewhere else?
- What could the STI clinic do to make testing for STI easier for you?
  - How do you think other sex workers feel about that?
- What care do you think the STI clinic now offers you as a sex worker?
- What care are you looking for at the STI clinic?
- What care do you expect at the STI clinic? What care are you missing?
- What do you think of the STI clinic’s current care offer?
  - What do other sex workers think about this?
- What do you know about PrEP?
  - In case participant doesn't know anything about PrEP 🡪 Would you like more information about this?
  - If participant knows anything about PrEP 🡪 What do you know about this?
  - If participant is already taking PrEP 🡪 What is your motivation? How do you obtain PrEP (general practitioner/STI clinic /informal) and do you follow the recommended checks?
  - Did you know that it can also be done via the STI clinic?
- Would you be interested in PrEP?
  - If so, would you also like to obtain PrEP care through the STI clinic?
  - How can the STI clinic reach male sex workers with their PrEP care?

**Reaching MSM sex workers**

Have you ever received a message from the STI clinic inviting you to come and test/get vaccinated?

- What did you think of this?
- Do you think this encourages other sex workers?

We would like to reach more men who have sex with men for money or goods with our STI care. Do you have any ideas on how we can do that best?

- Through which sites/places? Why there?
- With which means of communication? Apps/posters/folders/website? Why this one?
- With what message? Why?

How would you like to make an appointment and communicate with the STI clinic?

Do you know other men who do MSM sex work?

- Do you have contact with them?
- Would you be willing to advise fellow sex workers to take an STI test (give access to a test package with a card)?
- Can you contact someone with your questions or problems about sex work?
- Do you have any other ideas or tips about how we can reach you and your colleagues?

**STI and hepatitis B**

And we talked about STI and hepatitis B before. We want to come back to that.

Can you tell us a little bit about STI?

- Which STI are there? How can you get infected? What are consequences of an STI?

Suppose you have an STI: What consequences would that have for you?

- How bad would you that be for you?
- What do you think is the chance that you will get an STI?
- Do you feel like you're in control of getting an STI?
- Do you talk to your environment (friends and family) about STI?
- Do you know what your environment thinks of an STI?
- Do you know people who have had or still have an STI?
  - What do you think about that? Has that changed anything for you?

And can you tell us what you know about hepatitis B?

- What you know about hepatitis B?
  - How can you get infected?
  - What are the consequences?
  - How can you protect yourself?

Do you ever look for information about sexuality and sexual health?

- If yes: What kind of information are you looking for and where are you looking?
- Have you missed information / are you missing information as a (novice) sex worker?
- How can the STI clinic inform you of this? (Which communication channels? What means of communication?)

**Taboo and stigma sex work and MSM**

Do people around you know that you do this work?

- If yes:
  - What do they think of this? How did they react to this?
  - How important is their opinion to you/how much influence does their opinion have on you?
- What do you think your environment thinks about it?

When you took an STI test, did you tell them that you do sex work?

What's it like for you to do sex work?

- What positive thoughts or feelings do you have about yourself doing this work?
- What less pleasant thoughts or feelings do you have about yourself doing this work?

Do you ever notice that people react to you in a certain way or think about you through your work?

- Does the reaction of others about sex work influence whether or not you’re coming to the STI clinic for an STI test or vaccination?

Do you ever experience shame for the work?

- Has this ever stopped you from/is this stopping you from taking an STI test/asking for care?

Do people around you know that you have sex with men?

- What do you think your environment thinks of that?
- Do you ever notice that people around you react to you in a certain way or think about you because of this?
- Do you ever experience shame yourself because you have sex with men?
- Does the reaction of others to you having sex with men influence whether or not you’re coming to the STI clinic for an STI test or vaccination?

Closing questions

These were the questions we wanted to ask you. Are there any other things you'd like to share with us? Do you have any comments or comments about things we've discussed?
